# Successful Long-Term Enzyme Replacement Therapy in a Patient with Delayed-Onset ADA Deficiency

**DOI:** 10.1007/s10875-024-01794-7

**Published:** 2024-09-12

**Authors:** Vasil Toskov, Pawan Bali, Michael S. Hershfield, Stephan Ehl, Carsten Speckmann

**Affiliations:** 1 Division of Pediatric Hematology and Oncology, Department of Pediatrics and Adolescent Medicine, Medical Center, University of Freiburg, Faculty of Medicine, Freiburg, Germany; 2 Department of Medicine and Biochemistry, Duke University Medical Center, Durham, NC USA; 3 Institute for Immunodeficiency, Center for Chronic Immunodeficiency (CCI), Medical Center, Faculty of Medicine, University of Freiburg, Breisacher Str. 117, 79106 Freiburg, Germany

**Keywords:** ADA deficiency, Enzyme replacement therapy, Delayed-onset, Hypomorphic ADA

To the Editor

This letter summarizes our experience with exclusive long-term enzyme replacement therapy (ERT) in a now 20-year-old delayed-onset ADA deficient patient. Deficiency of the purine pathway enzyme adenosine deaminase 1 (ADA) is an inborn error causing severe combined immunodeficiency (SCID) due to elevated levels of deoxyadenosine nucleotides (dAXP), which are particularly toxic for maturing lymphocytes and thymic progenitor cells [1]. *ADA* null mutations give rise not only to SCID but also to various non-immunological manifestations (e.g. neurodevelopmental delay, sensorineural deafness, skeletal dysplasia), whereas patients with hypomorphic variants have residual ADA activity and develop a heterogeneous phenotype termed delayed-/late-onset ADA deficiency with variable degrees of immunodeficiency and immune dysregulation, and usually no relevant non-immunological abnormalities [2]. There are currently three treatment options for ADA deficiency: (i) hematopoietic stem cell transplantation (HSCT), (ii) autologous gene therapy (GT), and (iii) ERT. HSCT and GT are curative for the immunodeficiency and the only options allowing for full and long-term immune reconstitution in ADA-SCID. In ADA-SCID patients ERT is usually considered a valuable bridging treatment option, e.g. for improvement of lung disease, detoxification of the haematological niche and transient recovery of T cell lymphopenia, while the patient is prepared for HSCT or GT. ERT usually does not allow for a long-term full immune reconstitution in ADA-SCID, and compliance is critical for successful long-term therapy [3–5].

In contrast to ADA-SCID, treatment recommendations for delayed- or late-onset ADA patients are not clearly defined and the potential role for ERT remains unclear. Prospective data on the role of long-term ERT are limited to few case reports with individual patients, mostly with ADA-SCID [5], and this has been further complicated by the stop of production of ADAGEN^®^ (PEGylated bovine-extracted ADA) and its replacement with REVCOVI^®^ a PEGylated recombinant bovine ADA produced in E. coli, in 2018 [6].

Even though HSCT or GT are the only curative treatment options leading to complete immune reconstitution in patients with ADA-SCID [3, 4] no recommendations exist regarding long-term management of delayed- and late-onset ADA deficiency. Whether long-term ERT can be sufficient as a primary therapy in this group has not been studied in prospective trials and the published experience is difficult to interpret. Most reports on prolonged ADA-ERT are based on observations in ADAGEN^®^ treated patients and it remains difficult to compare these with observation in patients receiving primary or secondary REVCOVI^®^, i.e. in regards to a long-term sufficient immune reconstitution [4].

This letter illustrates two important aspects relevant for the treatment of delayed-onset ADA deficiency: (i) the potential feasibility of long-term immune reconstitution by exclusive ERT, and, (ii) the successful transition from ADAGEN^®^ to REVCOVI^®^ and its effect on the immunological and clinical phenotype. We analysed our treatment experience in a now 20-year-old patient with delayed-onset ADA deficiency, who has been on continuous ERT over the past 13 years. The initial clinical presentation of our patient has been reported previously [1]. In short, he presented at 7 years of age with lymphopenia, palmar/plantar warts and non-infectious interstitial lung disease (Supplementary Table 1). At the time of diagnosis, ADA activity in dried blood spots was barely detectable and dAXP levels were elevated at 7–9% of total adenine nucleotides (normal < 1%). Genetic testing revealed two pathogenic variants in *ADA* (c.780 + 1G > A, an intron 8 splice donor site mutation, and c.396dupA, p.Val133SerfsX38), compatible with the diagnosis of delayed-onset ADA deficiency. HSCT and GT were both discussed as the only curative non-experimental options, but were denied by the family. Therefore, intramuscular ADA ERT with ADAGEN^®^ was initiated and resulted in prompt resolution of pulmonary symptoms and good immune reconstitution. However, our patient developed neutralizing antibodies against the protein already after 4.5 months, which also was associated with a lack of enzyme activity (Fig. 1). However, treatment efficacy could be completely restored by short-term administration of intravenous immunoglobulin (IVIG) and increased, supraphysiological ADAGEN^®^ dosing [1]. For the following 9 years he sustained normal ADA activity under ADAGEN^®^ (dose 20–40 U/kg/week), which led to full and long-term detoxification, immune reconstitution, as well as sustained clinical and radiographic resolution of his interstitial alveolitis. Given the surprisingly uncomplicated clinical course, stable immune function and maintenance of full detoxification, the possibility of a somatic reversion in *ADA* was excluded by genetic reanalysis of sorted lymphocyte populations at age 16. After transition to REVCOVI^®^ in 2019 his lymphocyte counts initially decreased, similar to the observations by Murguia-Favela et al. on ADA-SCID patients transitioning to REVCOVI^®^ [6], without signs of clinical disease, a reduction of ADA activity or an increase of toxic %dAXP. Under continued REVCOVI^®^ ERT he has experienced neither increased infection susceptibility nor immune dysregulation. Moreover, despite fluctuating numbers of CD19 + cells, he has sustained normal immunoglobulin levels, positive specific antibody responses (Supplementary Table 1), and has not required immunoglobulin replacement therapy. Despite decreasing REVCOVI^®^ dose per week (initially 0.15 mg/kg/week, at last follow-up 0.14 mg/kg/week), the lymphocyte counts have recovered after the initial dip following REVCOVI^®^ initiation. Of particular importance is the observation that the number of CD4 + 45RA + naïve T cells also remained stable under sustained detoxification, which seems to reflects sufficient thymic output and function under ERT.

**Fig. 1:**
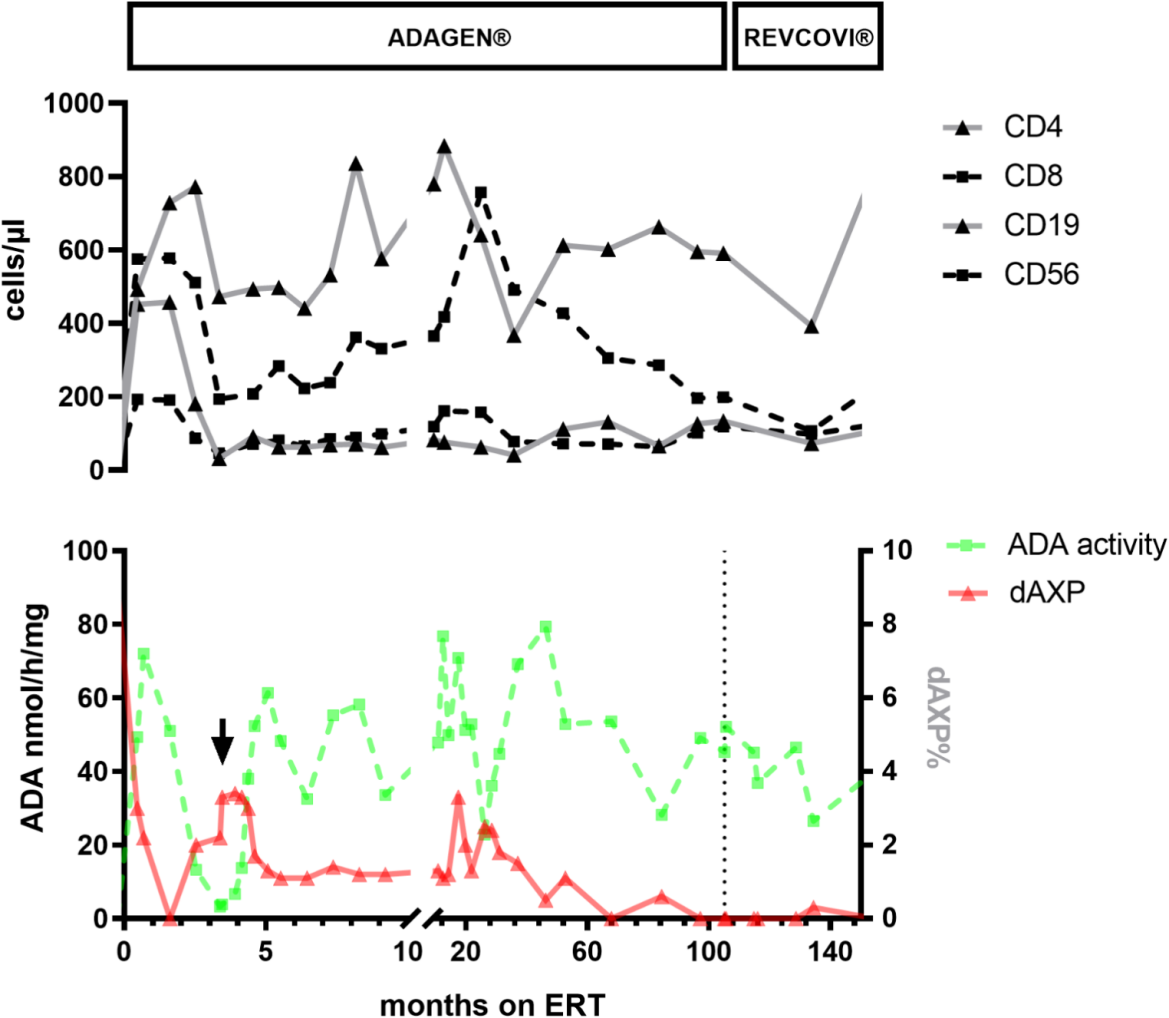
The course of lymphocyte subsets in relation to ADA activity and dAXP. Top panel: lymphocyte subset course determined via flow cytometry before and during ERT with ADAGEN^®^/REVCOVI^®^. Black line indicates CD4 + cells, spotted gray line CD8 + cells, gray line CD19 + cells, and gray dashed line CD16/56 + cells. Bottom panel: ADA activity (green dashed line) and total adenine deoxyribonucleotides (dAXP) levels (red line) as determined by the analysis of dried blood spots prepared with whole EDTA blood from the time point of diagnosis until 155 months after the initiation of ERT. The vertical arrow signifies the dose increase of ADAGEN^®^. Reference values > 18 years of age [10]: CD4 + cells (28–57%, 300–1400 counts/µl), CD8 + cells (10–39%, 200–900 counts/µl), CD19 + cells (6–19%, 100–500 counts/µl), CD56 + cells (7–31%, 90–600 counts/µl)

Seamless transition from ADAGEN^®^ to REVCOVI^®^ was feasible, and both immune phenotype and clinical disease have remained well controlled in our patient. Moreover, he has experienced no non-immunological complications (e.g. cognitive, behavioural, or neurological). Some disadvantages of ERT remain, such as high cost, need for regular intramuscular administration, and continuous monitoring of ADA activity and dAXP concentration. Moreover, neutralizing antibodies may lead to a loss of effectiveness, which also occurred in our patient during treatment with ADAGEN^®^, but could be salvaged via IVIG and ERT dosage increase. A further potential limitation of long-term ERT is decreasing treatment adherence. Decreased compliance over time has been described in ADA-SCID patients, where HSCT/GT was deferred due to successful ERT, and may lead to severe morbidity [5]. Re-initiation of ERT led to clinical and immunological improvement, even if long-term sequelae remained [5]. Importantly, long-term immune reconstitution in ADA-SCID patients on ERT usually remains incomplete [7], and is mainly used as a short-term detoxifying bridge to GT/HSCT [4, 6], as these remain the treatment of choice even in older patients [8]. Patients treated with HSCT or GT with decreasing graft function and increasing dAXP concentration may also benefit from ERT and achieve detoxification after transition from ADAGEN^®^ to REVCOVI^®^ [9].

In contrast to reports from ADA-SCID, we can show long-term normalization of the immune profile in our delayed-onset ADA patient under continuous ERT without any overt disease activity. Thus, our experience exemplifies the clinical value of exclusive, long-term ERT and the successful transition from ADAGEN^®^ to REVCOVI^®^ in a patient with delayed-onset ADA deficiency. Our report suggests that long-term ERT is feasible and can be an effective conservative treatment in patients with hypomorphic *ADA* variants, which is in stark contrast to the morbidity experienced by ADA-SCID patients on ERT [5, 7]. To explore the long-term effect of ERT on the clinical and immunological phenotype of patients with delayed-onset ADA deficiency, more prospective data are warranted, ideally in the setting of disease-specific registries.

## Electronic Supplementary Material

Below is the link to the electronic supplementary material.

Supplementary Material 1

## Data Availability

No datasets were generated or analysed during the current study.
