# Supplementary material for: Successful Long-Term Enzyme Replacement Therapy in a Patient with Delayed-Onset ADA Deficiency
Source: J Clin Immunol. 2024 Sep 12;45(1):8. doi: 10.1007/s10875-024-01794-7 (PMC11393103; doi:10.1007/s10875-024-01794-7)
Supplement: Supplementary file 1 — Supplementary Material 1 [file 10875_2024_1794_MOESM1_ESM.docx]

Supplementary Table 1: Laboratory investigations at initial diagnosis, and after initiation of ADAGEN® and REVCOVI®

|  | Initial | ADAGEN® | REVCOVI® |
| --- | --- | --- | --- |
| WBC [10^3^/µl] | 3,6 | 8300 | 6000 |
| Lymphocytes [10^3^/µl] | 972 | 1445 | 1190 |
| CD4+ [10^3^/µl] | 147 | 744 | 811 |
| CD8+ [10^3^/µl] | 535** | 297 | 225 |
| CD4+45RA+ [%] | 44 | 59 | 64 |
| CD4+45RA+ [µl] | 65 | 439 | 519 |
| CD19+ [10^3^/µl] | 12 | 140 | 106 |
| CD16/56+ [10^3^/µl] | 21 | 179 | 124 |
| IgM [mg/dl] | 14 | 69 | 88 |
| IgG [mg/dl] | 732 | 1122 | 1162 |
| IgA [mg/dl] | 43 | 219 | 274 |
| IgE [mg/dl] | high*** | 462 | 176 |
| Measles IgG | yes | no | nd |
| Rubella IgG | nd | no | yes* |
| Tetanus IgG | yes | yes | yes |
| SARS-CoV-2-S1-IgG |  |  | yes |
| Proliferation  -anti-CD3/-CD28  -PHA | reduced | yes | yes |
|  | reduced | yes | yes |
|  | no | yes | yes |
| CD: cluster of differentiation, Ig: immunoglobulin, PHA: phytohemagglutinin, WBC: white blood cells, *MMR booster in 2021, **two populations CD8low (γδ cells) and CD8high, ***not reported | | | |
